# Do citations and impact factors relate to the real numbers in publications? A case study of citation rates, impact, and effect sizes in ecology and evolutionary biology

**DOI:** 10.1007/s11192-012-0822-6

**Published:** 2012-08-28

**Authors:** Christopher J. Lortie, Lonnie W. Aarssen, Amber E. Budden, Roosa Leimu

**Affiliations:** 1 Department of Biology, York University, 4700 Keele St., Toronto, ON M3J 1P3 Canada; 2 Department of Biology, Queen’s University, Kingston, ON K7L 3N6 Canada; 3 National Centre for Ecological Analysis and Synthesis, Santa Barbara, CA 93101 USA; 4 Department of Plant Sciences, University of Oxford, South Parks Road, Oxford, OX1 3RB UK

**Keywords:** Citations, Ecology, Effect size, Evolutionary biology, Hypothesis testing, Treatments

## Abstract

Metrics of success or impact in academia may do more harm than good. To explore the value of citations, the reported efficacy of treatments in ecology and evolution from close to 1,500 publications was examined. If citation behavior is rationale, i.e. studies that successfully applied a treatment and detected greater biological effects are cited more frequently, then we predict that larger effect sizes increases study relative citation rates. This prediction was not supported. Citations are likely thus a poor proxy for the quantitative merit of a given treatment in ecology and evolutionary biology—unlike evidence-based medicine wherein the success of a drug or treatment on human health is one of the critical attributes. Impact factor of the journal is a broader metric, as one would expect, but it also unrelated to the mean effect sizes for the respective populations of publications. The interpretation by the authors of the treatment effects within each study differed depending on whether the hypothesis was supported or rejected. Significantly larger effect sizes were associated with rejection of a hypothesis. This suggests that only the most rigorous studies reporting negative results are published or that authors set a higher burden of proof in rejecting a hypothesis. The former is likely true to a major extent since only 29 % of the studies rejected the hypotheses tested. These findings indicate that the use of citations to identify important papers in this specific discipline—at least in terms of designing a new experiment or contrasting treatments—is of limited value.

## Introduction

The strength of quantitative analyses in most disciplines is self-evident. Nonetheless, it is reasonable to occasionally stop counting and crunching numbers and examine whether the process is enhancing or hindering the process of inquiry and discovery in a discipline. The general value of citations and impact factors is both vigorously discussed and often contested (Kokko and Sutherland 1999; Kotiaho 1999; Lawrence 2007; Monastersky 2005). From a purely qualitative or common sense perspective, this is quite reasonable. Authors may cite the work of others (or themselves) for a variety of reasons and this may vary from purely rationale to mostly haphazard (Aksnes and Rip 2009). The most compelling/amusing instance is the differential success of those with surname initials closer to the beginning of the alphabet securing tenure sooner in economics likely due to the higher citation frequency to their work (Einav and Yariv 2006) since papers in this field are given credit in alphabetic order. Studies may also be cited as positive examples of an effect or as negative examples due to poor execution, i.e. never test this system as done in a certain instance. Unfortunately, academics may also cite the papers of the people they know well or papers with bigwig coauthors (Leimu et al. 2008). Ultimately, none of this really matters unless it obscures our capacity to either appreciate the interest and utility of others work (Small 2004) or hinders our ability as a discipline to effectively conduct synthesis and discover important avenues of research. Hence, in this spirit of discovery, we examine whether the utility of research treatments or strength of biological effects predicts the citation rates to a given study.

Meta-analyses in ecology and evolutionary biology (EEB) are becoming increasingly common. These synthesis papers provide the perfect substrate to examine the relationship between citations and the efficacy of a treatment or strength of an ecological/biological effect within a discipline. Hence, we used the datasets from each study reported in published meta-analyses in EEB as a case study to examine the following predictions. (1) Studies with larger effect sizes should be more cited if authors use the work of others to identify the utility of successful treatment methods or to reference the strongest biological effects (Barto and Rillig 2012; Small 2004). (2) Higher impact journals should on average publish studies with larger effect sizes if they do indeed differentiate for stronger evidence (Song et al. 2000). (3) The effect sizes associated with accepting or rejecting a hypothesis (as concluded by the authors within a study) should be related to the effect since it is likely more difficult to publish negative results (Csada et al. 1996). Together, these predictions provide an assessment of the strength of citations as a proxy or shorthand for identifying studies that succeeded in testing a hypothesis. As an aside, this is not to imply that studies in EEB often with relative smaller effect sizes are unimportant—simply that we may need to be cautious in interpreting highly cited studies as important or as necessary indications of effective experimentation.

## Methods

Using the ISI Thompson Web of Science, a search was conducted for studies with the terms ‘meta-analysis’ and ‘ecology’ or ‘evolution’ (10/2011). Of the available literature, meta-analyses that both listed ISI-indexed studies used in the analyses and provided the effect size estimates for each study (or plots) were selected resulting in 39 appropriate meta-analyses for a total of 1,332 individual studies (“Appendix”). Synthetic studies were excluded if only grand means were reported. The impact factor of the journal associated with each publication and the total citations (excluding self-cites) to each at that point in time were recorded (also from Web of Science). Each study was then inspected to determine whether the authors concluded support or rejection of the hypothesis tested. Generalized linear models were used to test for an effect of reported effect size estimates on citations per year for each publication (fit to a Poisson distribution) and on impact factor at the journal level (exponential fit). Post hoc regressions were used to further examine statistically significant relationships or visualize data. The import of impact factor and effect size on conclusion to support or reject a hypothesis was tested with a generalized logistic model with appropriate post hoc contrasts. The sensitivity of topic to the outcome of analyses was also examined by iterative grouping of studies into sub-disciplines and there was no effect on the results reported herein. All statistics were done in JMP 9.02 (SAS 2012).

## Results

**Fig. 1:**
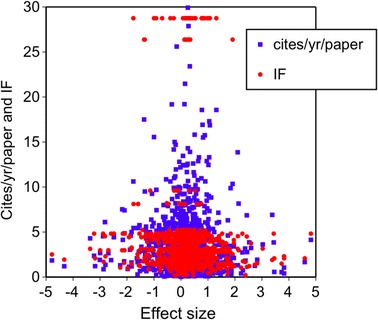
*Post hoc* visualizations of the importance of effect size within study on the citations per year per publication and the associated journal impact factors (*n* = 1,332, see text for GLM statistics)

The effect sizes reported within each study did not significantly affect the citations per year per paper (GLM, χ^2^ = 0.4, *p* = 0.53, *df* = 1; Fig. 1 depicted with blue squares) or impact factor (GLM, χ^2^ = 0.17, *p* = 0.68, *df* = 1; Fig. 1 depicted with red circles). The effect sizes within each study did however significantly relate to the decision to accept or reject the hypotheses tested (GLM, χ^2^ = 16.6, *p* = 0.0001, *df* = 1) with higher effect sizes associated with rejection—but only 29 % of the studies in this database reported rejecting the hypotheses examined.

## Discussion

**Fig. 2:**
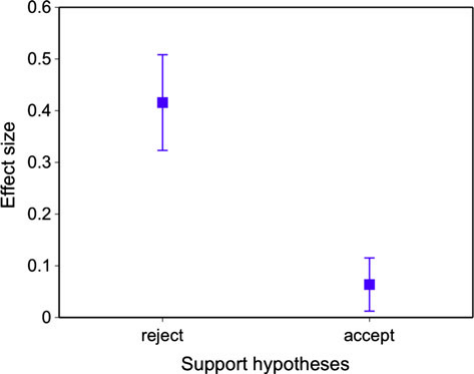
The mean effect sizes associated with the interpretation by the authors of each paper to accept (*n* = 951) or reject (*n* = 381) the hypotheses tested. The mean ± 1SE is depicted

A wide range of mechanisms could be directed towards testing the utility of citations in advancing discovery within an academic discipline. However, we proposed three direct predictions associated with their immediate capacity, at least within EEB, to explore this topic. The effect size of a given study did not directly predict its citation rate, and at a larger scale, populations of effect sizes associated with journals did not predict the impact factor of the journals. The findings associated with these two first predictions suggest that authors do not generally use the effect size of a given study (i.e. the effectiveness of the treatment or strength of ecological effect) when selecting studies to reference. Finally, the magnitude of the effect sizes was greater within studies that rejected the hypotheses tested. This final prediction is intriguing as it is either evidence supporting the file-drawer problem, i.e. studies with negative results are unpublished (Csada et al. 1996) or more difficult to publish, or indicates that authors set a much higher standard before choosing to interpret findings as failure to support a hypothesis. Taken together, these simple tests serve as a diagnostic tool in identifying whether authors within a specific discipline are on average citing the work of others via the strength of treatment effects reported or for other reasons. This approach has only been applied once in a similar manner, and relationships between effect size and impact factor and the file-drawer problem were also confirmed (Barto and Rillig 2012). The purpose of this other study was to explore the prevalence of dissemination biases in EEB, and the conclusions were similar in that theory tenacity and confirmatory citation behavior were detected. Barto and Rillig however detected a positive relationship between effect size and citation rates, and this varies from the current study for the following two reasons: positive effects were correlated with citations but negatives were not whereas in this study they did not vary when tested independently, and whilst the sample sizes between the two studies are equivalent, the search terms they used were much broader and included chemical ecology and global change. Consequently as one might expect, citation behavior varies dramatically even between sub-disciplines. It also indicates that more detailed reporting (i.e. effect sizes and other data quality measures in addition to *p* values) in EEB would be useful in promoting both primary synthesis and quantitative, scientometric discussions including more critical contrasts (Fig. 2)

Whilst the goal of EEB is to discover the natural world, pressing demands on the planet for a clear signal to the public on appropriate actions should encourage scientists to more effectively manage the evidence we both report and reference. Systematic reviews, meta-analyses, and short research forum papers all provide the necessary substrate for the balanced enhancement of the research process within a discipline (Roberts et al. 2006; Treadwell et al. 2007). The relative success of treatments, hypotheses, or methodologies can be identified (Lortie and Callaway 2006). More importantly, research gaps or avenues of productive research can also be defined. Consequently, citation behavior within specific experimental studies—not reviews—need not rigorously adopt and report how studies were selected to support the research described or the conclusions and interpretations proposed. Nonetheless, the visibility of certain studies commonly cited sets the benchmark and stage for subsequent discovery. The identification of important papers is thus not best done via citations although many journals now promote and highlight their most cited papers. We propose that this proxy for ‘hot’ papers be used sparingly since it fails on a per publication basis and at across journals as well. Admittedly, journals still likely serve as a useful means to organize papers for the time being both topically and by their relative efficacy in examining treatments and processes in EEB, but many other factors should be considered in categorizing papers as important in this field. Logistical constraints of experimentation on animals and low samples associated with capturing mobile organisms in EEB may lower effect size estimates but not their importance. Some topics are also important regardless of effect size estimates because they speak to critical issues such as conservation or restoration. Given that controversial papers can be more cited and also have higher effect sizes (or at least those that made it into print), a logical starting point would be to both select formal reviews that report selection criteria for studies synthesized and to identify individual published studies which *fail to support* the hypotheses tested. This hybrid approach of big picture synthesis and error complements many major developments in the epistemological study of science that proposes that error is the critical tool for advancement (Ford 2000; Ford and Ishii 2001; Mayo 1996). In EEB, surprises are also reportedly very common (Doak et al. 2008) and searching for and citing them may to some extent alleviate the file-drawer problem. This would accelerate discovery within our field via the avoidance of repetition of treatments and studies previously tested but ignored or largely unavailable. Finally, the art of discovery can certainly be achieved by a thorough analysis of all the pertinent facts but also by looking where others fail to. Individually examining why we cite the papers we do is thus necessary.

## Appendix

**Table:** The published meta-analyses in ecology and evolutionary biology that provided effect size estimates for ISI-indexed studies reported herein

Study ID	References
1	Arnqvist, G., & Nilsson, T. (2000). The evolution of polyandry: multiple mating and female fitness in insects. *Animal Behavior*, *60*, 145–164.
2	Bender, D.J., Contreras, T., & L. Fahrig. (1998). Habitat loss and population decline: a meta-analysis of the patch size effect. *Ecology*, *79*, 517–533.
3	Boissier, J., Morand, S., & Mone, H. (1999). A review of performance and pathogenicity of male and female *Schistosoma mansoni* during the life-cycle. *Parasitology*, *119*, 447–454.
4	Connor, E.F., Courtney, A.C., & Yoder, J.M. (2000). Individuals–area relationships: the relationship between animal population density and area. *Ecology*, *81*, 734–748.
5	Cote, I.M., & Poulin, R. (1995). Parasitism and group size in social animals: a meta-analysis. *Behavioral Ecology*, *6*, 159–165.
6	Cote, I.M., & Sutherland, W.J. (1997). The effectiveness of removing predators to protect bird populations. *Conservation Biology*, *11*, 395–405.
7	Curtis, P.S. (1996). A meta-analysis of leaf gas exchange and nitrogen in trees grown under elevated carbon dioxide. *Plant, Cell, and Environment*, *19*, 127–137.
8	Dolman, P.M., & Sutherland, W.J. (1997). Spatial patterns of depletion imposed by foraging vertebrates: theory, review and meta-analysis. *Journal of Animal Ecology*, *66*, 481–494.
9	Downing, J.A., Osenberg, C.W., & Sarnelle, O. (1999). Meta-analysis of marine nutrient-enrichment experiments: variation in the magnitude of nutrient limitation. *Ecology*, *80*, 1157–1167.
10	Englund, G., Sarnelle, O., & Copper, S.D. (1999). The importance of data-selection criteria: meta-analyses of stream predation experiments. *Ecology*, *80*, 1132–1141.
11	Fiske, P., Rintamaki, P.T., & Karvonen, E. (1998). Mating success in lekking males: a meta-analysis. *Behavioral Ecology*, *9*, 328–338.
12	Gontrad-Danek, M.C., & Moller, A.P. (1999). The strength of sexual selection: a meta-analysis of bird studies. *Behavioral Ecology*, *10*, 476–486.
13	Griffin, A.S., & West, S.A. (2003). Kin discrimination and the benefit of helping in cooperatively breeding vertebrates. *Science*, *302*, 634–636.
14	Huberty, A.F., & Denno, R.F. (2004). Plant water stress and its consequences for herbivorous insects: a new synthesis. *Ecology*, *85*, 1383–1398.
15	Hyatt, L.A., Rosenberg, M.S., Howard, T.G., Bole, G., Fang, W., Anastasia, J., Brown, K., Grella, R., Hinman, K., Kurdziel, J.P., & Gurevitch, J. (2003). The distance dependence prediction of the Janzen-Connell hypothesis: a meta-analysis. *Oikos*, *103*, 590–602.
16	Jennions, M.D., Moller, A.P., & Petrie, M. (2001). Sexually selected traits and adult survival: a meta-analysis. *The Quarterly Review of Biology*, *76*, 3–36.
17	Koricheva, J. (2002). Meta-analysis of sources of variation in fitness costs of plant antiherbivore defenses. *Ecology*, *83*, 176–190.
18	Koricheva, J., Larsson, S., Haukioja, E., Keinänen, M. (1998). Regulation of woody plant secondary metabolism by resource availability: hypothesis testing by means of meta-analysis. *Oikos*, *83*, 212–226.
19	Leimu, R., & Koricheva, J. (2006). A meta‐analysis of genetic correlations between plant resistances to multiple enemies. *The American Naturalist*, 168, e15–e37.
20	Leimu, R., & Koricheva, J. (2006). A meta-analysis of tradeoffs between plant tolerance and resistance to herbivores: combining the evidence from ecological and agricultural studies. *Oikos*, *112*, 1–9.
21	Leimu, R., Mutikainen, P., Koricheva, J., & Fischer, M. (2006). How general are positive relationships between plant population size, fitness and genetic variation? *Journal of Ecology*, *94*, 942–952.
22	Maestre, F., Valladares, F., Reynolds, J.F. (2005). Is the change of plant–plant interactions with abiotic stress predictable? A meta-analysis of field results in arid environments. *Journal of Ecology*, *93*, 748–757.
23	McCurdy, D.G., Shutler, D., Mullie, A., Forbes, M.F. (1998). Sex-biased parasitism of avian hosts: relations to blood parasite taxon and mating system. *Oikos*, *82*, 303–312.
24	Micheli, F. (1999). Eutrophication, fisheries, and consumer-resource dynamics in marine pelagic ecosystems. *Science*, *285*, 1396–1398.
25	Shykoff, J.A., & Moller, A.P. (1999). Fitness and asymmetry under different environmental conditions in the barn swallow. *Oikos*, *86*, 152–158.
26	Moller, A.P., & Alatalo, R.V. (1999). Good-genes effects in sexual selection. *Proceedings of the Royal Society of London B*, 266, 85–91.
27	Moller, A.P., & Thornhill, R. (1998). Bilateral Symmetry and Sexual Selection: A Meta‐Analysis. *The American Naturalist*, 151, 174–192.
28	Nykänen, H., & Koricheva, J. (2004). Damage-induced changes in woody plants and their effects on insect herbivore performance: a meta-analysis. *Oikos*, *104*, 247–268.
29	Poulin, R. (2000). Manipulation of host behaviour by parasites: a weakening paradigm? *Proceedings of the Royal Society of London B*, 267, 787–792.
30	Poulin, R. (2000). Variation in the intraspecific relationship between fish length and intensity of parasitic infection: biological and statistical causes. *Journal of Fish Biology*, *56*, 123–137.
31	Saikkonen, K., Lehtonen, P., Helander, M. Koricheva, J., & Faeth, S.H. (2006). *Trends in Plant Science*, *11*, 428–43.
32	Schmitz, O.J., Hamback, P.A., & Berckerman, A.P. (2000). Trophic cascades in terrestrial systems: a review of the effects of carnivore removals on plants. *The American Naturalist*, *155*, 141–153.
33	Sheridan, L.A.D., Poulin, R., Ward, D.F., & Zuk, M. (2000). Sex differences in parasitic infections among arthropod hosts: is there a male bias? *Oikos*, *88*, 327–334.
34	Sokolovska, N., Rowe, L., & Johansson, F. (2000). Fitness and body size in mature odonates. *Ecological Entomology*, *25*, 239–248.
35	Torres-Vila, L.M., & Jennions, M.D. (2005). Male mating history and female fecundity in the Lepidoptera: do male virgins make better partners? *Behavioral Ecology and Sociobiology*, *57*, 318–326.
36	Valkama, E., Koricheva, J., Oksanen, E. (2007). Effects of elevated O_3_, alone and in combination with elevated CO2, on tree leaf chemistry and insect herbivore performance: a meta-analysis. *Global Change Biology*, *13*, 184–201.
37	Vander Werf, E. (1992). Lack’s clutch size hypothesis: an examination of the evidence using meta-analysis. *Ecology*, *73*, 1699–1705.
38	Van Zandt, & Mopper, S. (1998). A meta‐analysis of adaptive deme formation in phytophagous insect populations. *The American Naturalist*, *152*, 595–604.
39	Vollestad, L., Hindar, K., Moller, A.P. (1999). A meta-analysis of fluctuating asymmetry in relation to heterozygosity. *Heredity*, *83*, 206–218.
